# Unique N-Terminal Interactions Connect F-BOX STRESS INDUCED (FBS) Proteins to a WD40 Repeat-like Protein Pathway in Arabidopsis

**DOI:** 10.3390/plants10102228

**Published:** 2021-10-19

**Authors:** Edgar Sepulveda-Garcia, Elena C. Fulton, Emily V. Parlan, Lily E. O’Connor, Anneke A. Fleming, Amy J. Replogle, Mario Rocha-Sosa, Joshua M. Gendron, Bryan Thines

**Affiliations:** 1 Instituto de Biotecnología, Universidad del Papaloapan, Tuxtepec 68301, Mexico; esepulveda@unpa.edu.mx; 2 Departamento de Biología Molecular de Plantas, Instituto de Biotecnología, Universidad Nacional Autónoma de México, Cuernavaca 62250, Mexico; rocha@ibt.unam.mx; 3 Biology Department, University of Puget Sound, Tacoma, WA 98416, USA; efulton@alumni.pugetsound.edu (E.C.F.); eparlan@alumni.pugetsound.edu (E.V.P.); l.e.oconnor@wustl.edu (L.E.O.); afleming@alumni.pugetsound.edu (A.A.F.); areplogle@pugetsound.edu (A.J.R.); 4 Department of Molecular, Cellular and Developmental Biology, Yale University, New Haven, CT 06511, USA; joshua.gendron@yale.edu

**Keywords:** F-box protein, SCF complex, stress response, WD40 repeat-like protein

## Abstract

SCF-type E3 ubiquitin ligases provide specificity to numerous selective protein degradation events in plants, including those that enable survival under environmental stress. SCF complexes use F-box (FBX) proteins as interchangeable substrate adaptors to recruit protein targets for ubiquitylation. FBX proteins almost universally have structure with two domains: A conserved N-terminal F-box domain interacts with a SKP protein and connects the FBX protein to the core SCF complex, while a C-terminal domain interacts with the protein target and facilitates recruitment. The F-BOX STRESS INDUCED (FBS) subfamily of plant FBX proteins has an atypical structure, however, with a centrally located F-box domain and additional conserved regions at both the N- and C-termini. FBS proteins have been linked to environmental stress networks, but no ubiquitylation target(s) or biological function has been established for this subfamily. We have identified two WD40 repeat-like proteins in Arabidopsis that are highly conserved in plants and interact with FBS proteins, which we have named FBS INTERACTING PROTEINs (FBIPs). FBIPs interact exclusively with the N-terminus of FBS proteins, and this interaction occurs in the nucleus. FBS1 destabilizes FBIP1, consistent with FBIPs being ubiquitylation targets SCF^FBS1^ complexes. This work indicates that FBS proteins may function in stress-responsive nuclear events, and it identifies two WD40 repeat-like proteins as new tools with which to probe how an atypical SCF complex, SCF^FBS^, functions via FBX protein N-terminal interaction events.

## 1. Introduction

At the onset of environmental stress, the ubiquitin 26S proteasome system (UPS) selectively degrades key cellular proteins to initiate plant responses that promote resilience and survival. Protein targets destined for removal are ubiquitylation substrates for E3 ubiquitin ligases, where one prevalent E3 ligase subtype is the SKP1-CUL1-F-box (SCF) complex [1]. SCF complexes use an interchangeable F-box (FBX) protein subunit as a substrate adaptor to specifically interact with unique protein targets [2,3,4,5]. FBX proteins almost universally have a structure with two domains: An N-terminal F-box domain facilitates interaction with a SKP protein and the core SCF complex, and a C-terminal domain interacts specifically with the target(s) [2]. This two-domain structure directly bridges core UPS components to precise protein targets under specific conditions, and it places FBX proteins at a dynamic interface that regulates diverse cellular pathways critical for plant life.

A very small number of FBX proteins, however, deviate from this typical two-domain protein structure. Many of these atypical FBX proteins have a centrally located F-box domain, a C-terminal target interaction domain, and an additional protein interaction domain at the N-terminus [6,7,8]. In humans, N-terminal domains can control subcellular localization [9], bind to an accessory protein that assists with C-terminal targeting events [10] or mediate regulatory interactions with other proteins [6,11,12]. The only plant FBX proteins with established N-terminal interaction dynamics belong to the ZEITLUPE (ZTL), FLAVIN-BINDING KELCH REPEAT F-BOX1 (FKF1), and LOV KELCH PROTEIN2 (LKP2) subfamily, which regulate the circadian clock and flowering time [8,13,14,15,16]. In addition to a central F-box domain, the ZTL/FKF1/LKP2 subfamily has a N-terminal blue-light sensing LOV domain and C-terminal kelch repeats [16], which are both used to recruit distinct ubiquitylation substrates [8,15,17,18]. The N-terminal LOV domain has additional roles that regulate the FBX function through an interaction with GIGANTEA (GI), which controls subcellular localization and protein stability [13,14]. Therefore, across kingdoms, a few atypical FBX proteins with a N-terminal protein interaction domain, in addition to a C-terminal targeting domain, achieve an expanded function by having further regulatory capacity and/or coordinating multiple cellular outputs through dual targeting.

F-BOX STRESS INDUCED (FBS) proteins constitute a far less understood subfamily of plant FBX proteins with an atypical structure [19,20,21]. Arabidopsis FBS1 is the founding member of this subfamily and is noteworthy for its broad biotic and abiotic stress-inducible gene expression profiles [19,21]. In FBS1, a centrally located F-box domain is flanked by two conserved regions present at the N- and C-termini, which do not match any known protein interaction domains or motifs [19]. FBS1 interacts with Arabidopsis SKP1 (ASK1) and can auto-ubiquitylate [19,20], suggesting that it forms a functional SCF-type E3 ligase in vivo. At least five of 13 Arabidopsis 14-3-3 regulatory proteins bind to FBS1 [20]. However, since this interaction requires both the N-terminal region and the F-box domain of FBS1 [20], and ubiquitylation presumably requires an unhindered F-box domain to interact with the SKP subunit of the SCF complex [1], the 14-3-3 proteins are unlikely ubiquitylation targets. Furthermore, an inducible *FBS1* gene construct had no discernable effect on FBS1 interactor 14-3-3λ protein abundance [20]. Importantly though, FBS1-interacting 14-3-3 proteins are negative regulators of Arabidopsis responses to cold and salt stress [22,23,24,25,26], which demonstrates another important link between FBS1 and environmental stress response networks in plant cells.

A more complete understanding of the FBS family protein function in plants has been stymied by two primary limitations. First, not knowing selective targeting relationships between SCF^FBS^ complexes and their putative substrates has left FBS action on cellular output pathways completely enigmatic. Second, functional redundancy within this family has likely thwarted past efforts seeking to establish a biological function. Arabidopsis *fbs1* plants have no obvious phenotype [19,21], however, three additional FBS family members that may be functionally redundant are encoded in the genome. Here, we identify two highly conserved WD40 repeat-like proteins that interact with multiple FBS family members in Arabidopsis, which we have named FBS INTERACTING PROTEINs (FBIPs). Interactions between all four FBS subfamily members and FBIP proteins occur in the nucleus, and interactions occur exclusively via the N-terminal domain of FBS proteins. These findings connect a stress network involving FBS proteins to nuclear processes, and they provide new tools with which to probe unique N-terminal interactions in FBX proteins in the context of plant stress responses.

## 2. Results

### 2.1. FBS Protein Interaction with ASK1

**Figure 1:**
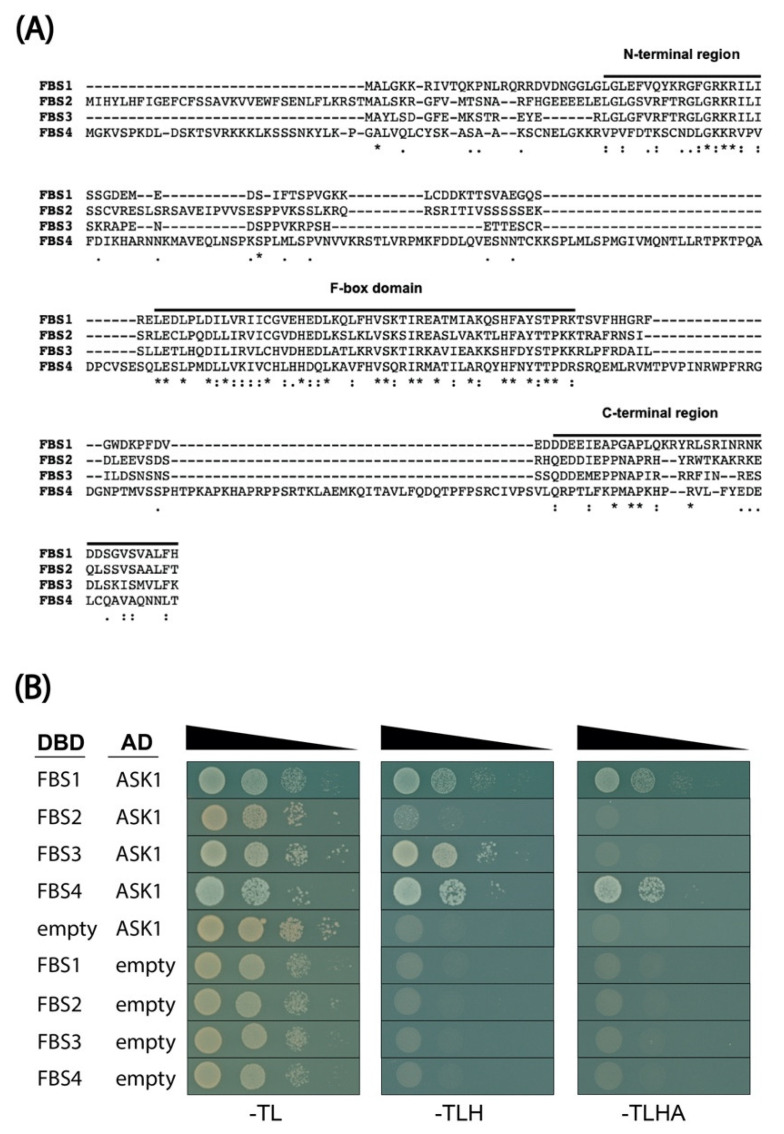
The Arabidopsis F-BOX STRESS INDUCED (FBS) protein family. (**A**) Full-length protein sequence alignment of the four Arabidopsis FBS family members (FBS1–FBS4) created with the T-COFFEE sequence alignment program. Asterisks are fully conserved residues, colons are strongly conserved residue properties, and periods are weakly conserved residue properties. (**B**) FBS family interactions with ASK1 in yeast two-hybrid assays. Diploid yeast strains with indicated test constructs as bait (DBD) and prey (AD) were grown in liquid culture, diluted (OD_600_ = 10^0^, 10^−1^, 10^−2^, 10^−3^), and spotted on SD medium minus Trp/Leu (-TL), minus Trp/Leu/His (-TLH), and minus Trp/Leu/His/Ade (-TLHA).

FBS1 is the founding member of a four-member FBX protein subfamily (FBS1–FBS4) in Arabidopsis. FBS2–FBS4, similar to FBS1, share a non-canonical structure with a centrally located F-box domain and conserved regions at their N- and C-termini (Figure 1A). The conserved region at the N-termini of FBS proteins spans approximately 20 residues, while the conserved region at the C-terminus encompasses about 35 residues (Figure 1A). FBS1 interacts with ASK1 and auto-ubiquitylates, indicating that FBS1 likely participates in functional SCF complexes [19,20]. However, the ability of other FBS family members to interact with ASK proteins remains unknown, as does the possibility of functional redundancy among family members. To interrogate this possibility, all four FBS family members were tested as bait constructs (DBD, GAL4 DNA-binding domain) for the interaction with ASK1 as prey (AD, GAL4 activation domain) under less stringent (-TLH) and more stringent (-TLHA) nutritional selection. Interactions were apparent between all four FBS family members on -TLH, although only very minimal growth was observed for FBS2 (Figure 1B). Only the interactions between FBS1 and FBS4 with ASK1 were apparent under the most stringent selection (-TLHA) (Figure 1B). Since Arabidopsis has 21 ASK proteins, it is possible that the FBS proteins showing minimal partnering with ASK1 interact more strongly with the other untested ASKs [27]. These interactions show, however, that FBS2–FBS4 are viable candidates for functional SCF complex substrate adapters, similar to FBS1.

### 2.2. Identification of a New FBS1 Interactor

**Figure 2:**
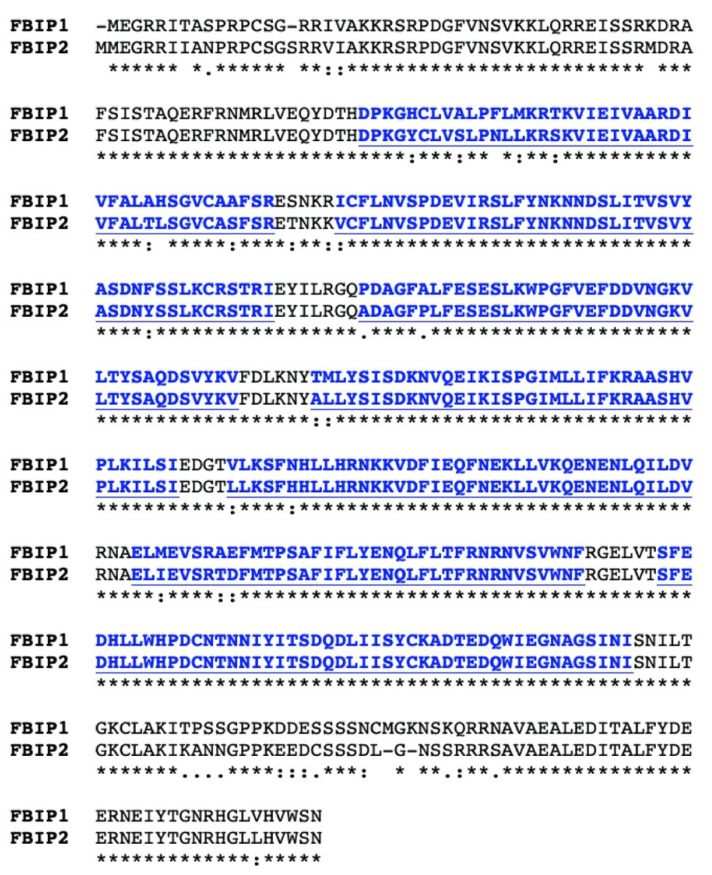
FBS INTERACTING PROTEIN (FBIP) sequence features. Full-length protein sequence alignment of the two Arabidopsis FBIP family members created with the T-COFFEE sequence alignment program. Blue indicates locations of seven WD40-like repeat sequences predicted by the WD40-repeat protein Structure Predictor version 2.0 (WDSPdb 2.0). Asterisks are fully conserved residues, colons are strongly conserved residue properties, and periods are weakly conserved residue properties.

In addition to ASK1, the only established FBS1 interacting proteins belong to the 14-3-3 family [20]. However, since the interaction dynamics are not consistent with ubiquitylation of 14-3-3 proteins by SCF^FBS1^ [20], we sought additional FBS1 interactors as candidate targets that could connect FBS proteins to biological processes. Two additional related proteins were identified as partners for FBS1, which we have named FBS INTERACTING PROTEINs (FBIPs). FBIP1 (At3g54190) was identified in the same yeast two-hybrid screen that found 14-3-3 proteins as FBS1 interactors [20]. FBIP1 is also listed as an FBS1 interactor by the SUBA4 database (http://suba.live/, accessed on 16 September 2021) from high-throughput protein-protein interaction (PPI) screening [28,29]. FBIP1 is 467 residues in length and is a member of the transducin/WD40 repeat-like superfamily of proteins. WD40 repeats typically form a β-propeller domain that acts as a scaffold in mediating protein-protein or protein-DNA interactions [30]. Seven putative WD40 repeat-like sequences were predicted in FBIP1 by the WD40-repeat protein Structures Predictor database version 2.0 (WDSPdb 2.0) [31], although these predictions fall into the low confidence category (Figure 2). A second protein highly similar to FBIP1 was identified in the Arabidopsis genome by BLAST search, which we have named FBIP2 (At2g38630). The protein sequence identity and similarity between FBIP1 and FBIP2 are just over 91% and 96%, respectively (Figure 2).

We gained no additional insight on the FBIP function using various bioinformatics resources. Other than putative WD repeat-like sequences, no sequence features were identified using various domain or motif prediction programs. BLAST and PSI-BLAST searches with FBIP1 and FBIP2 sequences failed to identify additional significant hits in Arabidopsis. We did, however, find very highly conserved FBIP protein sequences throughout the plant kingdom, including in bryophytes (the top BLAST hit in *Physcomitrella patens* is about 77% identical and 85% similar to *Arabidopsis* FBIP1). By investigating AtGenExpress ATH1 array datasets [32,33,34], we found that *FBIP1* is constitutively expressed in most tissues and organs of Arabidopsis, and throughout its life cycle, but we found no conditions where *FBIP1* is more highly expressed compared to the other conditions. *FBIP2* is not represented on the ATH1 array.

### 2.3. FBS Interactions with FBIPs

**Figure 3:**
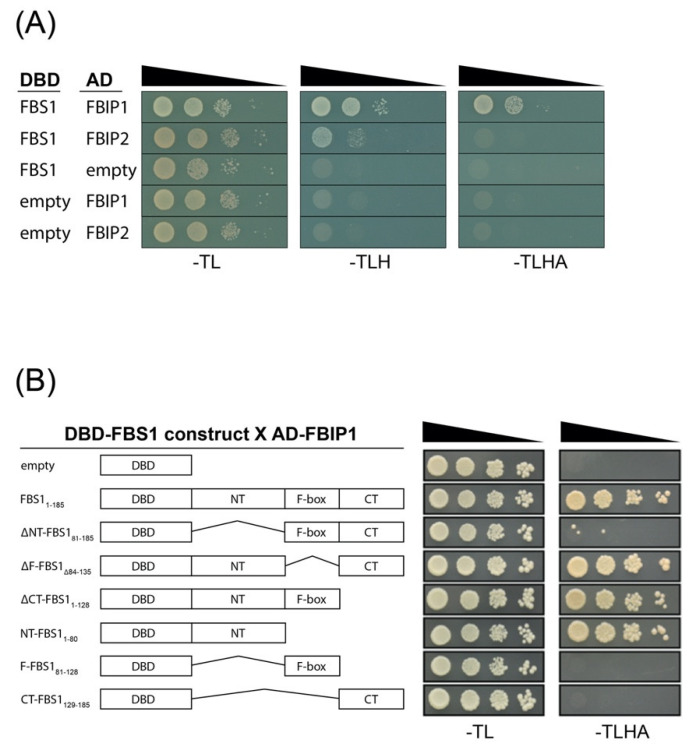
Yeast two-hybrid (Y2H) interactions between FBS1 and FBIP proteins. (**A**) Full-length FBS1 interactions with full-length FBIP1 and FBIP2. Diploid yeast strains with indicated test constructs as bait (DBD) and prey (AD) were grown in liquid culture, diluted (OD_600_ = 10^0^, 10^−1^, 10^−2^, 10^−3^), and spotted on SD medium minus Trp/Leu (-TL), minus Trp/Leu/His (-TLH), and minus Trp/Leu/His/Ade (-TLHA). (**B**) Truncated FBS1 bait (DBD) construct interaction with full length FBIP1 prey (AD). Amino acid deletions are indicated on the left.

We confirmed that the full-length FBS1 and FBIP1 interact with yeast two-hybrid analysis. The interaction between FBS1 and FBIP1 elicited growth in yeast strains on both less stringent (-TLH) and more stringent (-TLHA) nutritional selection, and FBS1 yielded growth with FBIP2 on -TLH (Figure 3A). Family-wide interactions between each FBS protein and the two FBIP proteins were also assessed (Figure S1). Growth was observed for FBS3 and FBIP1, but not with FBS2 or FBS4. No additional interactions were observed with FBIP2. Collectively, the yeast two-hybrid results suggest that FBS1 and FBIP1 might be the primary FBS/FBIP protein interaction pair or possibly bind with the strongest affinity, but that some other family-wide interactions might be possible.

FBS proteins have two regions of unknown function outside of the F-box domain and, presumably, at least one of these interacts with a target. In order to determine which parts of FBS1 are important for the FBIP1 interaction, we created truncated versions of FBS1 with the N-terminal (NT), F-box or C-terminal (CT) regions removed in different combinations and tested under stringent (-TLHA) selection (Figure 3B). Removing the N-terminal region (ΔNT-FBS1_81–185_) abolished the ability of FBS1 to interact with FBIP1, while removal of the F-box domain (ΔF-FBS1_Δ84–135_) or C-terminal region (ΔCT-FBS1_1–128_) did not. The FBS1 N-terminal region (NT-FBS1_1–80_) in combination with the full-length FBIP1 yielded growth on -TLHA, indicating that the FBS1 N-terminal domain alone is sufficient to mediate this interaction.

Near the conserved N-terminal domains of FBS1 and FBS2 we found a LXLXL sequence (Figure 1A), which is the most prominent form of an EAR motif found in many different types of transcriptional regulators [35,36]. The EAR motif mediates the interaction with the WD40 repeat-containing protein TOPLESS (TPL) and TOPLESS RELATED (TPR) co-repressor proteins [37,38,39]. We considered whether this LXLXL sequence in the N-terminal region of FBS1 might: (1) Function as a canonical EAR motif to interact with TOPLESS, and/or (2) if it could be important for mediating interactions with FBIPs. However, substituting all three leucine residues for alanine in FBS1 did not alter its interaction with FBIP1, and FBS1 did not interact with TPL (both as bait or as prey) in our yeast two-hybrid system.

### 2.4. FBS Interactions with FBIP Occur in the Nucleus

**Figure 4:**
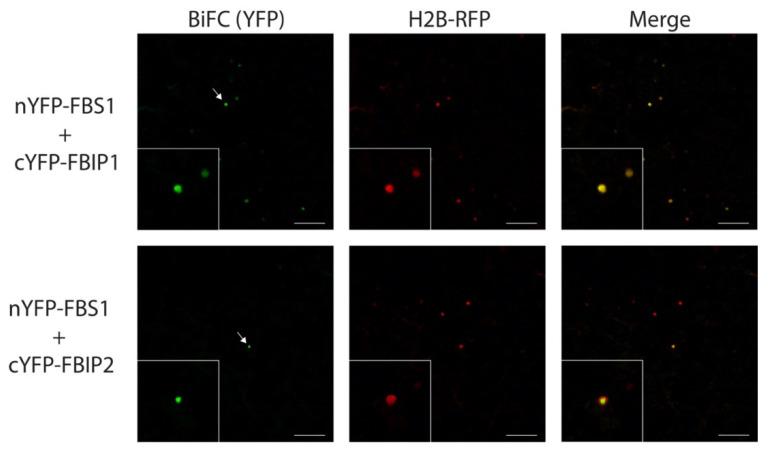
Bimolecular fluorescence complementation (BiFC) interactions between FBS1 and FBIP proteins. Laser-scanning confocal microscopy of *N. benthamiana* epidermal cells expressing N-terminal nYFP- or cYFP-tagged FBS1 and FBIP proteins. FBS1 interactions with FBIP1 (top row) or FBIP2 (bottom row) are visualized on the BiFC yellow channel (YFP, left column). A co-expressed H2B-RFP (as nuclear marker) is visualized on the red channel (RFP, middle column) and YFP/RFP images are overlaid (Merge, right column). Arrow indicates selected nuclei in the expanded inset image. Scale bar = 100 µm.

Next, we used bimolecular fluorescence complementation (BiFC) to test the FBS interaction with FBIP in plants and determine where the interaction occurs in a cell. The FBS and FBIP family proteins were expressed in *Nicotiana benthamiana* leaves as C-terminal fusions to either N-terminal (nYFP) or C-terminal (cYFP) halves of yellow fluorescent protein (YFP). In multiple independent experiments, the YFP fluorescence was observed for pairings between FBS1 and FBIP1 and FBIP2 (Figure 4). This YFP signal co-localized with that of a co-infiltrated H2B-RFP construct, which localizes exclusively in the nucleus [40], and shows that interactions between FBS1 and FBIP proteins also occur in the nucleus. Similar experiments found that FBS2–FBS4 also interact with FBIP1 in the nucleus (Figure S2). We observed interactions for FBS3 and FBS4 with FBIP2 (Figure S3), although we note that these interactions were more variable in the number of YFP positive nuclei across independent replicates, and with consistently fewer interactions for FBS3 and FBIP2. We did not observe any interactions between FBS2 and FBIP2. All FBS and FBIP fusion protein constructs were tested as pairs with empty nYFP or cYFP vectors, and in all pairings we were unable to detect any fluorescent signal similar to the FBS/FBIP test pairs (Figure S4). These findings show that in plants the FBS proteins participate in family-wide interactions in the nucleus.

### 2.5. FBS1 Destabilizes FBIP1

**Figure 5:**
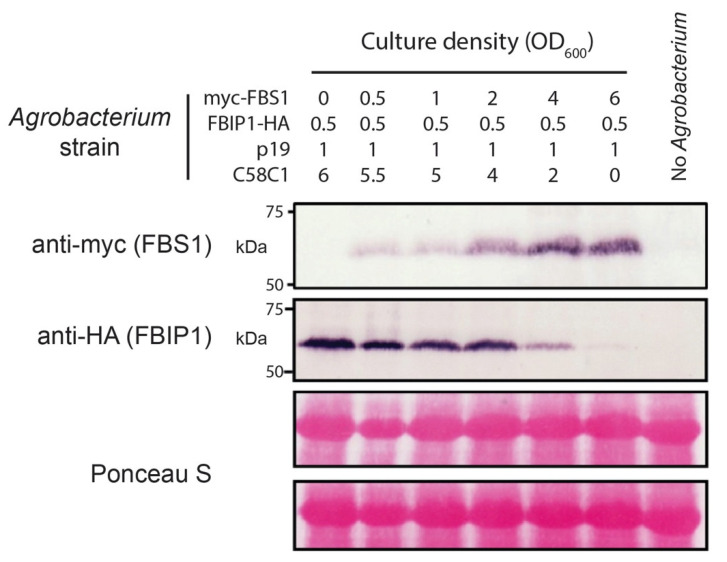
FBS1 influence on FBIP1 protein abundance in plants. *N. benthamiana* leaves were infiltrated with *Agrobacterium* (C58C1) strains to express the tagged proteins. *Agrobacterium* mixes contained varying cell densities of strains harboring expression constructs (myc-FBS1 and/or FBIP1-HA), a suppressor protein (p19) or untransformed cells. Total protein was isolated from leaves 3 days after infiltration, separated by SDS-PAGE, transferred, and probed with antibodies against myc (top row, FBS1) or HA (second row, FBIP1). Bottom two rows show Ponceau S staining of the major subunit of Rubisco from the same two blots as a loading control.

With the interaction established between multiple FBS and FBIP protein pairs, we next asked if the protein abundance relationship between FBS1 and FBIP1 is consistent with FBIP1 being a ubiquitylation target of SCF^FBS1^. If a protein is ubiquitylated by a particular SCF complex and subsequently degraded by the 26S proteasome, then increasing the abundance of the F-box component typically increases in vivo targeting and decreases substrate abundance [41]. Therefore, we tested the effects of varying FBS1 protein levels on the FBIP1 abundance in our *N. benthamiana* expression system by co-infiltrating *Agrobacterium* harboring these test constructs in different relative concentrations. Increasing the presence of FBS1 protein resulted in a corresponding decrease in the FBIP1 protein abundance by Western blot analysis (Figure 5). In comparison, when the FBS1 abundance was increased relative to the co-infiltrated 14-3-3λ in an identical setup, we did not observe any decrease in 14-3-3λ abundance as the amount of expressed FBS1 was increased (Figure S5). This finding is congruous with previous observations that FBS1 and 14-3-3 interactions are not consistent with targeting [20]. Therefore, since the abundance of FBIP1 decreases in an FBS1-dependent manner, we conclude that FBIPs are viable candidates for SCF^FBS1^ ubiquitylation targets.

## 3. Discussion

As substrate adapters for SCF-type E3 ligases, FBX proteins act at the interface between core UPS components and specific cellular outputs, including those that help plant cells mitigate the effects of environmental stress. Previous work with FBS1 strongly alluded to some role in plant stress responses, possibly by regulating the expression of stress genes [19,20,21], but a more detailed understanding was limited by the unknown identity of ubiquitylation target(s) and by possible redundancy within the *FBS* gene family. Here, we have identified a pair of WD40 repeat-like superfamily proteins, FBIP1 and FBIP2, that both interact with FBS family proteins. These family-wide interactions indicate that functional redundancy within these two families is likely, but at the same time suggest a more robust stress response module. The FBS protein interaction with FBIPs in the nucleus points to a role for these proteins in the regulation of gene expression and/or other chromosomal events. Finally, FBIP proteins are strong candidates for SCF^FBS^ ubiquitylation targets that act in plant stress responses, and they provide new tools with which to investigate unique FBX protein N-terminal events in plants.

The exclusive nuclear localization FBS and FBIP protein interactions under the conditions we tested offer a critical clue as to the molecular functions of both protein families. One hypothesis for the FBIP function stemming from this result is that they regulate gene expression, which is an idea supported by the finding that hundreds of JA/ABA and other stress genes are mis-expressed in the *fbs1-1* background [21]. Some plant nuclear localized WD40 repeat proteins have direct actions in transcription regulation [39,42,43] or chromatin modification [44,45,46], and in these cases the WD40 repeat proteins are essential components of multi-protein assemblies. For example, TOPLESS (TPL) is a well-studied WD40 repeat-containing co-repressor protein that acts in diverse developmental and environmental-response pathways [39]. TPL interacts with different DNA-bound transcriptional complexes and it recruits chromatin modifying enzymes and/or Mediator to repress gene expression [40,47,48]. TRANSPARENT TESTA GLABRA 1 (TTG1), another WD40 repeat protein, serves as a scaffold and mediates different combinations of bHLH and R2R3-type MYB DNA-binding transcription factors to regulate flavonoid metabolism and various developmental processes [43,49]. The FBIP proteins may function similarly to TPL or TTG1 and act as scaffolds and/or in recruitment roles for complexes that regulate transcription. Knowing additional FBIP interactors, which may include more recognizable proteins with readily inferred functions, will help address this hypothesis.

Future work will also be guided by questions that address interaction dynamics between FBIPs and the N-terminal region of FBS proteins, and the consequences of these associations. There are 13 residue positions in the FBS N-terminal region, ranging from moderately to absolutely conserved, that could be critical for the interaction with FBIPs. Future work will include identification of the exact residue or residues in FBS proteins mediating this interaction. Given numerous FBS connections to stress, but that *FBIP1* appears to be constitutively expressed across different plant organs and environmental conditions, it could be the case that FBIP proteins are components of a stress-response system working at the post-translational level. Next steps include a rigorous assessment of conditions under which SCF^FBS^ complexes form and interact with FBIP proteins in vivo. Furthermore, whether some additional factor (i.e., post-translational modification) stimulates SCF^FBS^ association with FBIP proteins, as in the case of some other SCF targeting events [50], is well worth investigating. The idea that additional in vivo factors or modification mediates the FBS/FBIP interaction is consistent with the finding that we observed more family-wide interactions in plant BiFC experiments compared to yeast two-hybrid. Knowing that SCF complexes in some atypical contexts ubiquitylate targets via the FBX protein N-terminal interactions [8,17], and that FBS1 appears to destabilize FBIP1, a leading hypothesis for future work is that FBIP proteins are bona fide ubiquitylation substrates for SCF^FBS^. Considering our work here and the general knowledge surrounding SCF action, our current model is that stress stimulates increased SCF^FBS^-dependent ubiquitylation of FBIP proteins, which are then degraded in response to this environmental trigger, resulting in cellular changes.

The atypical structure of FBS proteins, along with the identification of FBIPs as FBS N-terminal interactors, leads to a few intriguing hypotheses regarding how this SCF complex may impact cellular pathways in plant stress. If FBIP is a bona fide target with a biological function distinct from a more typical C-terminal target, then SCF^FBS^ complexes provide an exciting opportunity to study how plants coordinate more than one cellular pathway related to stress. N- and C-terminal targeting events might be simultaneous under a given condition, and in this situation SCF^FBS^ may integrate a response by ubiquitylating two distinct protein types, each interacting with a different region of the FBS substrate adapter. Alternatively, N- and C-terminal targeting may be asynchronous and condition dependent, in which case SCF^FBS^ may entail a switch that works in or leads to two different cellular states. At this point, however, we cannot completely exclude the possibility that FBIPs are not targets (see above), but instead serve in an alternative capacity that enables or inhibits the FBS action. One idea then is that FBIPs are accessories that help recruit other proteins as ubiquitylation targets. In humans, Cks1 directly associates with the N-terminus of FBX protein Skp2 to direct SCF^Skp2^ interaction with ubiquitylation target p27 in human cell cycle regulation [10,50]. In Arabidopsis, KAI2 and D14 interact with FBX protein MAX2 in SCF^MAX2^ complex to mediate ubiquitylation of SMXL transcription factors [51], though in these cases KAI2 and D14 are also FBX C-terminal interactors. To address these scenarios or others a critical piece of information to learn is the identity of a FBS C-terminal region-interacting protein that we presume to exist. Future work can then investigate higher order SCF^FBS^ complex assembly and action.

The 14-3-3 proteins directly regulate a range of cellular processes in plant cells [52], including core signaling pathways and transcriptional reprogramming events in cold and salt stress responses [22,23,24,25,26]. FBS protein interactions with FBIPs will almost certainly be a vital tool used to fully understand the connections between FBS proteins and the 14-3-3 protein regulatory network. Five of 13 Arabidopsis 14-3-3 proteins interact with FBS1. However, 14-3-3 proteins are unlikely ubiquitylation targets of SCF^FBS1^ and the consequences of these interactions are unknown [20]. One hypothesis regarding this interaction is that 14-3-3 proteins promote dimerization of SCF^FBS^ ligases [20], which in other situations enhances ubiquitylation targeting by SCF complexes [53,54]. As our understanding further develops regarding FBIPs as putative targets, their cellular abundance will be an essential readout in studies that investigate 14-3-3 effects on SCF^FBS^ activity. The 14-3-3 proteins exert regulatory effects through other mechanisms, however, through controlling the subcellular localization of client proteins or by shifting the location themselves [52]. In salt stress, FBS1 interactors 14-3-3λ and 14-3-3κ act at the plasma membrane and release signaling component SOS2 to activate salt stress tolerance [26,55]. Cold temperature triggers the FBS1 interactor 14-3-3λ to translocate from the cytosol into the nucleus where it interacts with and adjusts cold-responsive C-repeat-binding factor (CBF) action [25]. Considering that the FBS1 interaction with FBIPs was exclusively nuclear under the conditions tested here, an investigation of temporal and spatial aspects of 14-3-3/FBS interactions relative to FBS/FBIP interactions in plant cells before and during environmental stress will add more broadly to our understanding of the 14-3-3 stress response network in plant cells.

## 4. Materials and Methods

Bioinformatics: Gene and protein sequences were obtained from The Arabidopsis Information Resource (http://www.arabidopsis.org, accessed on 16 September 2021). Protein sequences were aligned using T-COFFEE (http://www.ebi.ac.uk/Tools/msa/tcoffee, accessed on 16 September 2021) accessed through the European Bioinformatics Institute (EBI) website (http://www.ebi.ac.uk, accessed on 16 September 2021) [56]. WD40 repeat-like sequences were identified in FBIP1 and FBIP2 using the WD40-repeat protein Structures Predictor database version 2.0 (WDSPdb 2.0; http://www.wdspdb.com/wdsp/, accessed on 16 September 2021) [31]. Basic Local Alignment Search Tool (BLAST) and Position-Specific Iterative (PSI)-BLAST were accessed through the National Center for Biotechnology Information (NCBI) website (http://www.ncbi.nlm.nih.gov, accessed on 16 September 2021) and used to search the RefSeq database. Candidate protein interactors were identified by searching the SUBA4 database (http://suba.live/, accessed on 16 September 2021) [29].

Gateway cloning: Gene-specific primers (Supplementary Table S1) were used with PCR to amplify coding sequences from pooled *Arabidopsis thaliana* (accession Col-0) cDNA. Amplicons were inserted into the pENTR/D-TOPO vector (Thermo Fisher Scientific, Waltham, MA, USA) according to the manufacturer’s protocols. Then, the genes were transferred with the LR Clonase II enzyme mix (Thermo Fisher Scientific, Waltham, MA, USA) into pCL112 or pCL113 [57] destination vectors for BiFC experiments, and into pGBKT7-GW (Addgene plasmid #61703) or pGADT7-GW (Addgene plasmid #61702) destination vectors for yeast two-hybrid experiments. Alternatively (Figure 3B), *FBS1* and *FBIP1* sequences were cloned into pBI770/pBI771 and tested for the interaction, as done previously [20]. Primers used to create *FBS1* truncation constructs are indicated in Supplementary Table S1.

Yeast two-hybrid assays: *Saccharomyces cerevisiae* cells were grown, transformed, mated, and selected by standard yeast protocols. Bait constructs (GAL4 DNA-binding domain, DBD) were transformed into Y2H Gold and prey constructs (GAL4 activation domain, AD) and Y187 strains by the LiAc method (Takara Bio; San Jose, CA, USA). Haploid strains were mated to produce diploid strains to test for the interactions. Diploid strains were grown for 24 h at 30 °C in the liquid synthetic defined (SD) medium minus Trp/Leu (-TL) medium with shaking. Thereafter, cells were washed in sterile water, cell concentrations were adjusted to OD_600_ = 10^0^, 10^−1^, 10^−2^, 10^−3^, and 10 μL was spotted on SD -TL (control), SD minus Trp/Leu/His (-TLH), and SD minus Trp/Leu/His (-TLHA) selective plates. The plates were incubated for 2 days at 30 °C and then scanned to produce images.

Bimolecular fluorescence complementation (BiFC): Recombinant plasmids were transformed into the *Agrobacterium tumefaciens* strain GV3101 (pMP90) by electroporation and selected under appropriate antibiotics. *A. tumefaciens* seed cultures were grown in LB with the appropriate antibiotic selection for 2 days with shaking at 30 °C. Then, they were used to inoculate 50 mL LB containing the appropriate antibiotics plus 10 μM acetosyringone and grown for an additional 24 h. The cells were pelleted and resuspended in the infiltration medium (10 mM MES, 10 mM MgCl_2_, 100 μM acetosyringone) and incubated for 5 h with rocking at room temperature. The cells were pelleted a second time, resuspended in the infiltration medium, and the appropriate nYFP/cYFP, H2B-RFP constructs were combined at a final OD_600_ of 1.0 for each test/control construct with suppressor strains (p19, γβ, PtoHA, HcPro) at a final OD_600_ of 0.5. *Nicotiana benthamiana* leaves from 4-week-old plants were infiltrated by a syringe with the *A. tumefaciens* mixes. The underside of whole leaf mounts was visualized using laser-scanning confocal microscopy 3 days after infiltration with a Nikon D-Eclipse C1 Confocal laser scanning microscope (Nikon Instruments) with either: (1) Excitation at 488 nm with an emission band pass filter of 515/30 or (2) excitation at 561 nm with an emission band pass filter of 650 LP.

Co-infiltration: *FBS1*, *FBIP1*, and *14-3-3λ* were cloned into pN-TAPa (9X myc tag), pGWB14 (3X HA tag) or pGWB12 (VSVG tag) vectors [58], respectively, using a Gateway strategy as above. Recombinant plasmids were transformed by electroporation into the *A. tumefaciens* strain C58C1Rif/pGV2260. *A. tumefaciens* was grown to a stationary phase in the LB medium containing the appropriate antibiotics plus 50 μg/mL acetosyringone. Bacteria were pelleted and washed with 10 mM MgCl_2_, and then resuspended in 10 mM MgCl_2_ and 150 μg/mL acetosyringone. Cell densities were adjusted to OD_600_ of 0.5. After 3 h of incubation, *A. tumefaciens* strains containing each construct were adjusted to varying concentrations and mixed with the same volume of an *A. tumefaciens* strain containing the viral suppressor p19, treated in the same way, but adjusted to OD_600_ of 1.0. The abaxial side of leaves from 3–4 week-old *N. benthamiana* were infiltrated with this bacterial suspension. After 3 days, the leaf material was collected and immediately frozen in liquid N_2_ for protein extraction.

Protein extraction and Western blotting: Approximately 100 μg of frozen tissue was homogenized in 200 μL of 1× Laemmli loading buffer plus 4 M urea, boiled for 5 min, and centrifuged at 10,000× *g* for 5 min. Then, 10 μL of the supernatant were loaded onto 8%, 10% or 15% polyacrylamide gels and subjected to SDS-PAGE using the standard protocols. The separated proteins were blotted onto a Hybond-P+ membrane (Amersham Pharmacia Biotech, Amersham, UK) using the standard protocols, and then the membranes were probed with anti-c-Myc, anti-HA antibody or anti-VSVG antibodies (all from Sigma-Aldrich, St. Louis, MO, USA). The blots were developed using an alkaline phosphatase kit (BCIP/NBT kit; Invitrogen; Waltham, MA, USA).

AGI numbers: FBS1 (At1g61340), FBS2 (At4g21510), FBS3 (At4g05010), FBS4 (At4g35930), FBIP1 (At3g54190), and FBIP2 (At2g38630).

## Data Availability

Not applicable.

## Supplementary Materials

The following are available online at https://www.mdpi.com/article/10.3390/plants10102228/s1, Figure S1: Yeast two-hybrid FBS1–FBS4 interactions with FBIP1 and FBIP2. Figure S2: Bimolecular fluorescence complementation (BiFC) interactions between FBS1–FBS4 and FBIP1. Figure S3: Bimolecular fluorescence complementation (BiFC) interactions between FBS1–FBS4 and FBIP2. Figure S4: YFP channel positive and negative controls. Figure S5: FBS1 influence on 14-3-3 λ protein abundance in plants. Table S1: Primer sequences used for cloning.
