# Supplementary material for: Unique N-Terminal Interactions Connect F-BOX STRESS INDUCED (FBS) Proteins to a WD40 Repeat-like Protein Pathway in Arabidopsis
Source: Plants (Basel). 2021 Oct 19;10(10):2228. doi: 10.3390/plants10102228 (PMC8537223; doi:10.3390/plants10102228)
Supplement: Supplementary file 1 [file plants-10-02228-s001.zip › Sepulveda-Garcia_supplemental_material.pdf]

SUPPLEMENTAL MATERIAL

Table S1. Primer sequences used for cloning

Function	Name	Sequence (5' → 3')
Gateway cloning for yeast two-hybrid and BiFC	FBS1_5'	CACCATGGCATTGGGGAAGAAAAGAATCG
	FBS1_3'	TCAGTGGAATAGAGCCACTGAGAC
	FBS2_5'	CACCATGATCCATTATCTCCATTTCA
	FBS2_3'	TCATGTAAACAAAGCCGCAG
	FBS3_5'	CACCATGGCGTATTTGAGTGATG
	FBS3_3'	TCATTTAAACAATACCATAGAGATCTTCGACAAATC
	FBS4_5'	CACCATGGGGAAGGTATCTCCAAAG
	FBS4_3'	TCAGGTGAGGTTGTTTTGAGC
	FBIP1_5'	CACCATGGAAGGAAGAAGGATTAC
	FBIP1_3'	TCAATTGGACCACACATGAAC
	FBIP2_5'	CACCATGATGGAAGGAAGAAGAATCAT
	FBIP2_3'	TCAATTAGACCAGACATGGAG
Cloning for yeast two-hybrid (Figure 3B)	SalFBS1_5'	TGCAGG**GTCGAC**ATATGGCATTGGG
	NotFBS1_3'	GTATCGATA**GCGGCCGC**TCAGTGGAATA
	SalFBS1 _5'	TGCAGG**GTCGAC**ATATGGCATTGGG
	NotNtFBS1_3'	A**GCGGCCGC**TCACTCTCGACTCTGACC
	SalFFBS1_5'	C**GTCGAC**TTGAAGATCTTCCCCTAGATA
	NotFFBS1_3'	CC**GCGGCCGC**TCAGGTGTACTATACGC
	SalCtFBS1_5'	C**GTCGAC**GTACACCTCGGAAAACCTCGG

SUPPLEMENTAL FIGURE LEGENDS

Figure S1. Yeast two-hybrid FBS1 – FBS4 interactions with FBIP1 and FBIP2. FBS2 – FBS4 family interactions with FBIP1 and FBIP2 in yeast two-hybrid assays. Diploid yeast strains with indicated test constructs as bait (DBD) and prey (AD) grown in liquid culture, were diluted ($OD_{600} = 10^0, 10^{-1}, 10^{-2}, 10^{-3}$), and spotted on SD medium minus Trp/Leu (-TL), minus Trp/Leu/His (-TLH), and minus Trp/Leu/His/Ade (-TLHA).

Figure S2. Bimolecular fluorescence complementation (BiFC) interactions between FBS1 – FBS4 and FBIP1. Laser-scanning confocal microscopy of *N. benthamiana* epidermal cells expressing N-terminal nYFP- or cYFP-tagged FBS and FBIP1 proteins. FBS2 (top row), FBS3 (middle row), and FBS4 (bottom row) interactions with FBIP1 are visualized on BiFC yellow channel (YFP, left column). A co-expressed H2B-RFP (as nuclear marker) is visualized on red channel (RFP, middle column) and YFP/RFP images are overlaid (Merge, right column). Arrow indicates selected nuclei in expanded inset image. Scale bar = 100 μm.

Figure S3. Bimolecular fluorescence complementation (BiFC) interactions between FBS1 – FBS4 and FBIP2. Laser-scanning confocal microscopy of *N. benthamiana* epidermal cells expressing N-terminal nYFP- or cYFP-tagged FBS and FBIP1 proteins. FBS2 (top row), FBS3 (middle row), and FBS4 (bottom row) interactions with FBIP2 are visualized on BiFC yellow channel (YFP, left

column). A co-expressed H2B-RFP (as nuclear marker) is visualized on red channel (RFP, middle column) and YFP/RFP images are overlaid (Merge, right column). Arrow indicates selected nuclei in expanded inset image. Scale bar = 100 µm.

Figure S4. YFP channel positive and negative controls. Laser-scanning confocal microscopy of *N. benthamiana* epidermal cells expressing GFP behind a CaMV 35S promoter (left), or nYFP- and cYFP fusion protein test constructs in combination with empty vectors. Note that image second from the right is that same as in Figure 4, for comparison, and the image on the far right is the same image as in Figure 4 but zoomed out. Scale bar in all images = 100 µm.

Figure S5. FBS1 influence on 14-3-3 λ protein abundance in plants. *N. benthamiana* leaves were infiltrated with *Agrobacterium* (C58C1) strains to express tagged proteins. *Agrobacterium* mixes contained varying cell densities of strains harboring expression constructs (myc-FBS1 and/or 14-3-3 λ-VSVG), a suppressor protein (p19), or untransformed cells. Total protein was isolated from leaves three days after infiltration, separated by SDS-PAGE, transferred, and probed with antibodies against myc (top row, FBS1) or VSVG (second row, 14-3-3 λ). Bottom two rows show Ponceau S staining of the major subunit of Rubisco from the same two blots as a loading control.
