# Supplementary figures and images for: Unique N-Terminal Interactions Connect F-BOX STRESS INDUCED (FBS) Proteins to a WD40 Repeat-like Protein Pathway in Arabidopsis
Source: Plants (Basel). 2021 Oct 19;10(10):2228. doi: 10.3390/plants10102228 (PMC8537223; doi:10.3390/plants10102228)

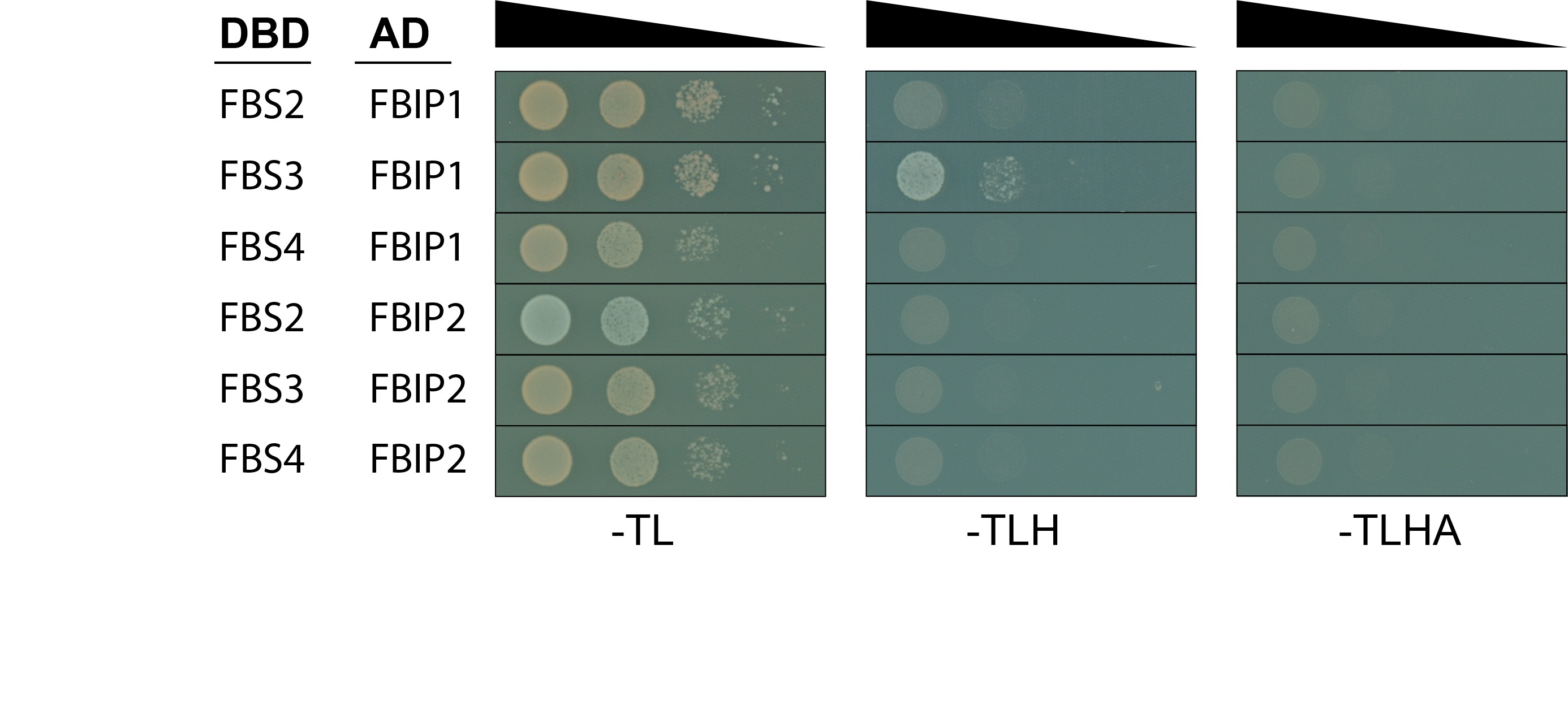

Supplement: Supplementary file 1 [file plants-10-02228-s001.zip › Figure S1.jpg]

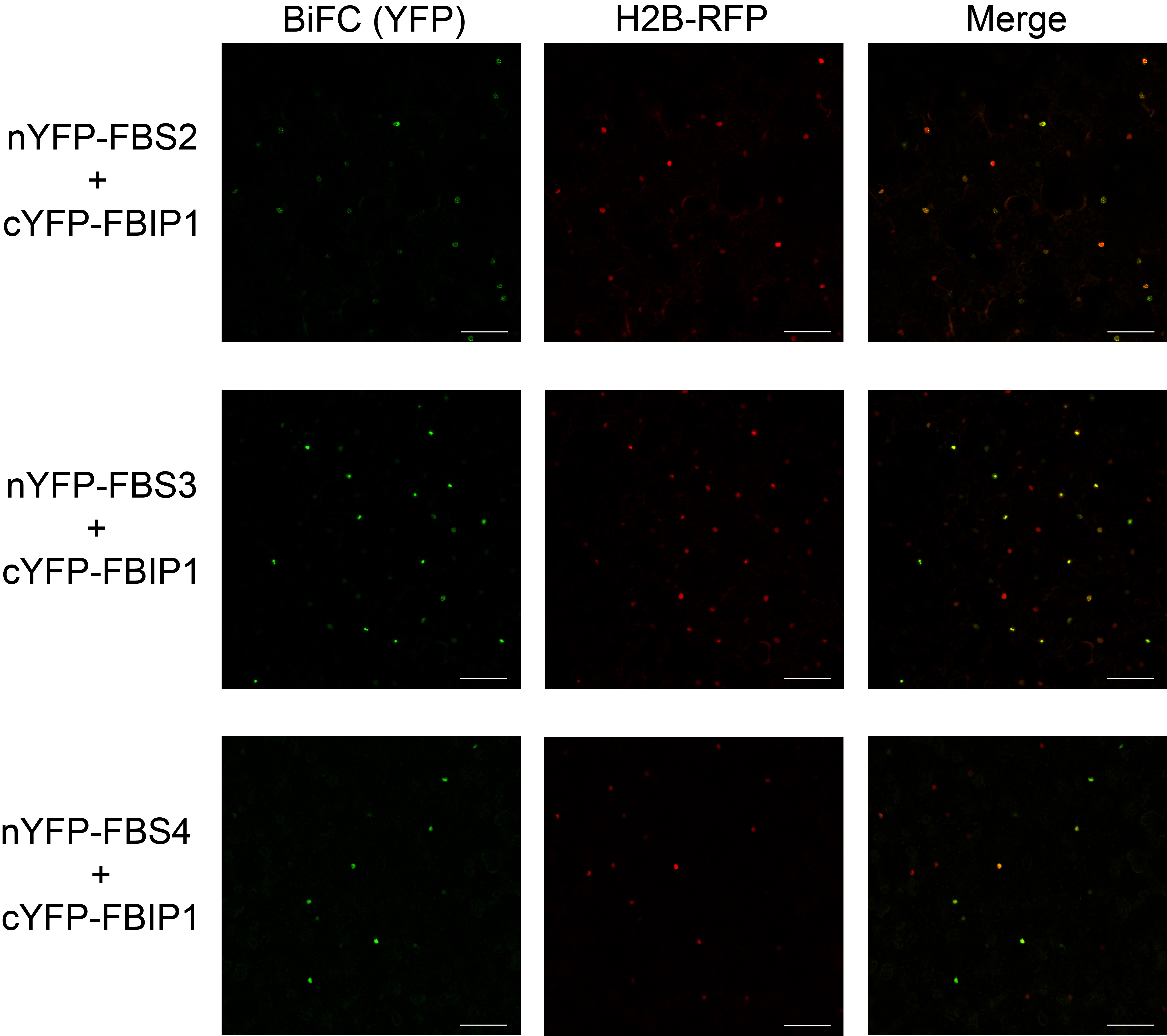

Supplement: Supplementary file 1 [file plants-10-02228-s001.zip › Figure S2.jpg]

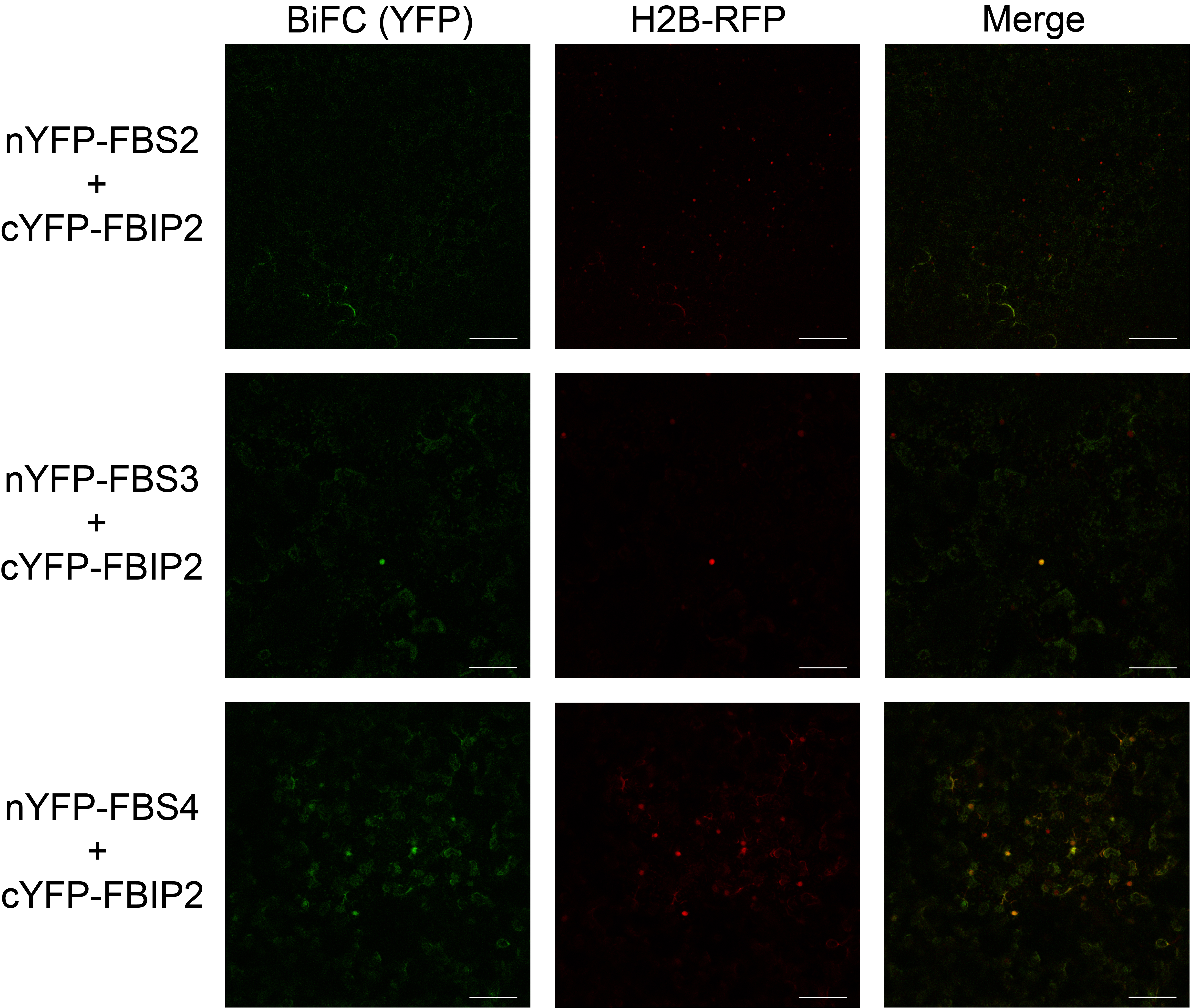

Supplement: Supplementary file 1 [file plants-10-02228-s001.zip › Figure S3.jpg]

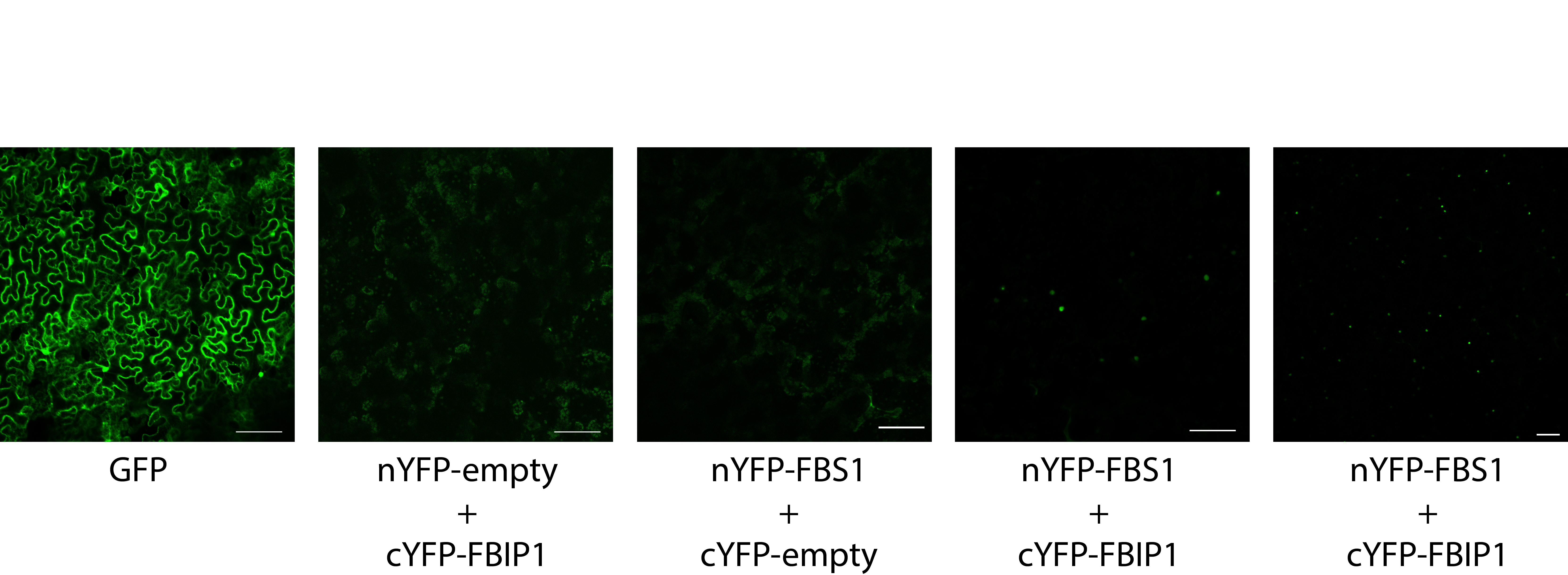

Supplement: Supplementary file 1 [file plants-10-02228-s001.zip › Figure S4.jpg]

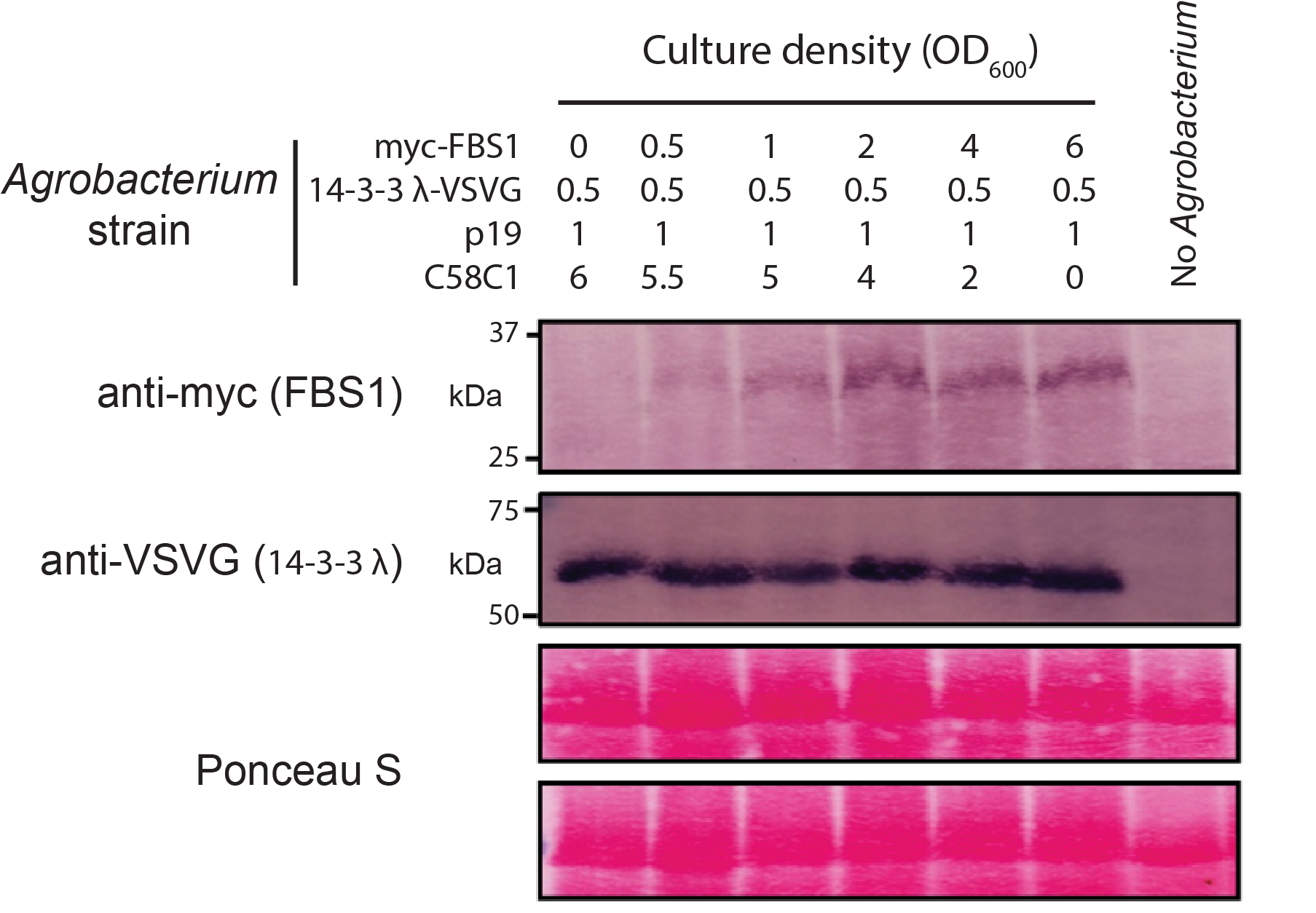

Supplement: Supplementary file 1 [file plants-10-02228-s001.zip › Figure S5.jpg]
